# Data about modification of structural and physicochemical properties of palm kernel expeller dietary fibres with carboxymethylation, acidic treatment, hydroxypropylation and enzymatic hydrolysis combined with heating

**DOI:** 10.1016/j.dib.2020.106285

**Published:** 2020-09-04

**Authors:** Yajun Zheng, Yan Li, Hailong Tian

**Affiliations:** College of Food Science, Shanxi Normal University, Linfen, 041004, China

**Keywords:** Palm kernel expeller dietary fiber, Acidic treatment, Carboxymethylation, Hydroxypropylation, Heating followed by enzymatic hydrolysis, Physicochemical properties

## Abstract

The data presented in this article are related to the research article entitled “Effects of carboxymethylation, acidic treatment, hydroxypropylation and enzymatic hydrolysis combined with heating on structural and physicochemical properties of palm kernel expeller dietary fibres.” This article describes the effects of carboxymethylation, acidic treatment, hydroxypropylation and enzymatic hydrolysis combined with heating on the structural and physicochemical properties of palm kernel expeller dietary fibres (PKEDF). Our data is made publicly available to the potential re-use of palm kernel expeller in food and other industries. Moreover, this dataset provides a reference about how to improve physicochemical and functional properties of dietary fiber.

## Specifications Table

**Table:** 

Subject	Physics, Chemistry
Specific subject area	Physicochemical and functional properties of dietary fiber
Type of data	Table, image (x-ray), text file, graph, figure
How data were acquired	Survey (a NS800 spectrocolorimeter, Shenzhen 3NH TECHNOLGOY CO. LTD., China; a Laser Diffraction Particle Size Analyzer, MS3000, Malvern instruments Ltd., UK), SEM: JSM-7500F scanning electron microscope, EOL, Tokyo, Japan, X-ray diffractometer (Ultima IV-185, Rigaku, Japan), Fourier-transformed infrared spectroscopy (660-IR FTIR spectrometer (Varian, USA). Emulsion capacity: FJ200-S Homogenizer, Hangzhou Qiwei Co., China. Viscosity: RVA-4 viscometer, RVA-TecMaster Co., Shanghai, China.
Data format	*Raw, filtered*
Parameters for data collection	PKE, palm kernel expeller; PKEDF, palm kernel expeller dietary fiber; PKEDF-A, PKEDF treated by acid; PKEDF-HE, PKEDF treated by enzymatic hydrolysis combined with heating; PKEDF-C, carboxymethylated PKEDF; PKEDF-H, hydroxypropylated PKEDF
Description of data collection	Description of how these data were collected is given in Experimental Design, Materials and Methods’ section
Data source location	Institution: College of Food Science, Shanxi Normal University City: Linfen Country: China
Data accessibility	The data are available with this article
Related research article	Author's names: Yajun Zheng, Yan Li, Hailong Tian Title: Effects of carboxymethylation, acidic treatment, hydroxypropylation and heating combined with enzymatic hydrolysis on structural and physicochemical properties of palm kernel expeller dietary fiber Journal: LWT - Food Science and Technology DOI: https://doi.org/10.1016/j.lwt.2020.109909

## Value of the Data

The data provide the potential re-use of palm kernel expeller in the food or other industries.
The data provide information on how to improve some functional properties of palm kernel expeller dietary fiber.
This data may serve as a reference for other works to develop other dietary fiber resource unutilized.

## 1 Data Description

**Fig. 1:**
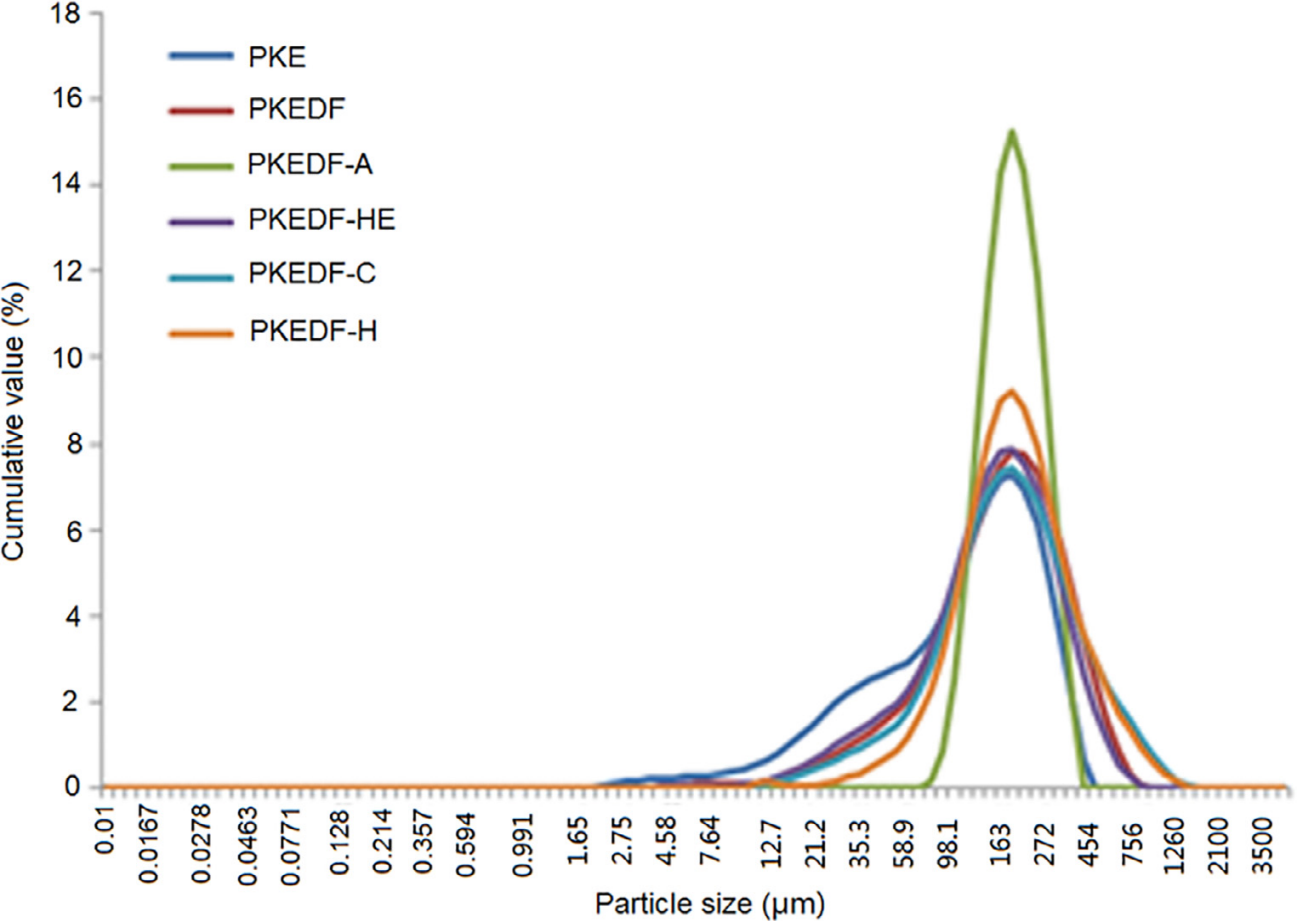
Particle size distribution of PKEDF and the modified PKEDFs.

The Fig. 1 shows the particle size distribution of PKE, PKEDF and the modified PKEDFs (PKEDF-A, PKEDF-HE, PKEDF-C and PKEDF-H). The data referring to water swelling capacity, emulsifying capacity, emulsion stbility, amylolysis kinetics and α-amylase activity inhibition ration (α-AAIR) of DFs are shown in Table 1, Table 2, Table 3, repscetiviely. The data referring to X-ray diffraction, Fourier-transformed infrared spectroscopy can be seen in the Ref. [1].

**Table 1:** Water swelling capacity of palm kernel expeller dietary fibres.

	Weight of dried DFs			Volume of dried DFs			Volume of DFs after the hydration		
PKE	0.545	0.5474	0.5432	2.8	2.9	2.4	3.3	3.6	3.1
PKEDF	0.5509	0.5458	0.5528	2.8	2.7	2.8	3.2	3.4	3.3
PKEDF-A	0.5504	0.5379	0.5466	2.6	2.5	2.6	3.1	2.9	2.9
PKEDF-HE	0.5452	0.5487	0.5471	2.7	2.9	2.8	3.8	4.1	3.9
PKEDF-C	0.5452	0.5487	0.5471	3.5	3.4	3.5	4.8	5.1	5.2
PKEDF-H	0.5456	0.531	0.5377	3	2.8	3.1	4.6	4.4	4.5

**Table 2:** Emulsion properties of palm kernel expeller dietary fibres.

DFs	Absorbance at 500 nm (A_0_)			Absorbance at 500 nm (A_t_)		
PKE	0.501	0.619	0.438	0.352	0.449	0.371
PKEDF	0.381	0.379	0.235	0.237	0.167	0.186
PKEDF-A	0.179	0.235	0.203	0.122	0.136	0.133
PKEDF-HE	0.373	0.399	0.447	0.267	0.264	0.248
PKEDF-C	0.669	0.766	0.715	0.564	0.674	0.696
PKEDF-H	0.655	0.654	0.611	0.476	0.487	0.465

**Table 3:** Effect of palm kernel expeller dietary fibres on amylolysis kinetics and α-amylase activity inhibition ration.

Time	Absorbance at 490 nm											
	Control			PKE			PKEDF			PKEDF-A		
20	0.286	0.291	0.315	0.237	0.283	0.204	0.17	0.182	0.162	0.143	0.097	0.131
40	0.517	0.601	0.589	0.339	0.366	0.361	0.294	0.334	0.33	0.209	0.229	0.244
60	0.77	0.685	0.681	0.622	0.626	0.613	0.588	0.577	0.484	0.442	0.499	0.504
80	0.816	0.792	0.846	0.699	0.706	0.702	0.693	0.677	0.629	0.679	0.554	0.651
100	0.947	0.965	0.913	0.813	0.821	0.814	0.77	0.845	0.846	0.72	0.723	0.696
120	1.077	1.001	0.997	0.955	0.907	0.941	0.871	0.88	0.893	0.831	0.978	0.887

Time	PKEDF-HE			PKEDF-C			PKEDF-H					
20	0.118	0.119	0.128	0.128	0.133	0.109	0.116	0.173	0.145			
40	0.161	0.178	0.189	0.256	0.173	0.291	0.262	0.252	0.256			
60	0.316	0.388	0.363	0.387	0.344	0.356	0.459	0.309	0.423			
80	0.506	0.406	0.492	0.53	0.539	0.537	0.488	0.474	0.596			
100	0.569	0.572	0.55	0.686	0.648	0.689	0.689	0.679	0.722			
120	0.774	0.677	0.717	0.838	0.788	0.788	0.721	0.791	0.748			

## 2 Experimental Design, Materials, and Methods

In the current study, effects of acidic treatment, carboxymethylation, hydroxypropylation and dual enzyme hydrolysis combination with heating on structral and physicochemical properties of palm kernel expeller dietary fibres (PKEDF) were studied. Firstly, PKEDF was prepared from defatted palm kernel expeller with α-amylase, alcalase and glucoamylase. Then PKEDF was modifed by acidic treatment (PKEDF-A), carboxymethylation (PKEDF-C), hydroxypropylation (PKEDF -H) and dual enzyme hydrolysis combination with heating (PKEDF-HE), respctively. The chemical composition, particle size distribution, color, structural and phsicochemical properties incuding water swelling capacity, oil holding capacity, emulsifying capacity, emulsion stability and α-amylase activity inhibition ration were studied [1, 5, 6].

### 2.1 Heating treatment followed by enzymatic hydrolysis

PKEDF (50 g) was heated at 121 °C for 45 min. The heated PKEDF was suspended in deionized water (dH_2_O) (1: 15, m/v), and then 0.3 g of hemicellulase and 0.45 g of cellulase were added. The mixture was adjusted to pH 4.5 and incubated at 50 °C for 3 h. Afterwards the mixture was incubated in boiling water for 10 min, and then cooled and filtered. The residue was collected and dried at 45 °C for 4 h to obtain PKEDF treated by heating with enzymatic hydrolysis (PKEDF-HE).

### 2.2 Acidic treatment

1 mol/L NaOH (1: 10, w/v) at 60 °C for 2 h, and then treated by 1 mol/L HCl at 60 °C for 30 min. Afterwards, the mixture was neutralized and filtrated, the residue was washed with dH_2_O and dried at 60 °C for 4 h. Then PKEDF treated by acidic (PKEDF-A) was obtained [2].

### 2.3 Carboxymethylation

PKEDF (8 g) were suspended in 80 mL of ethanol (85%, v/v). The suspension was gently stirred at room temperature (RT) for 30 min. Then 50 mL of NaOH (0.676 mol/L) were gradually added with vigorously stirring at 35 °C for 60 min. Afterwards 9.7 mL of chloroacetic acid (3.38 mol/L) pre-treated with NaOH (3.38 mol/L) were added and gently stirred at 35 °C for 30 min. Then the mixture was incubated at 53 °C for 3.5 h, and cooled to RT, neutralized to pH 7.0 with acetic acid and centrifuged at 4000 *g* for 15 min. The precipitate was collected, washed with anhydrous ethanol and dried at 45 °C for 4 h to obtain carboxymethylated PKEDF (PKEDF-C) [3].

### 2.4 Hydroxypropylation

5 g of PKEDF were suspended in 50 mL of dH_2_O and then mixed with 50 mL of Na_2_SO_4_ (20 mg/mL). The mixture was adjusted to pH 11.0 and 2 mL of propylene oxide were added. The mixture was stirred (180 r/min) at 40 °C for 24 h, and then filtrated and the residue was collected and dried at 45 °C for 8 h [4].

### 2.5 Structural properties

The scanning electron microscopy (SEM), Fourier-transformed infrared spectroscopy, and X-ray diffraction (XRD) analysis of DFs were performed using the same procedure as described by Zheng & Li [2].

## Declaration of Competing Interest

The authors declare that they have no known competing financial interests or personal relationships which have, or could be perceived to have, influenced the work reported in this article.

## Appendix Supplementary materials

Image, application 1
